# Explainable AI for Bipolar Disorder Diagnosis Using Hjorth Parameters

**DOI:** 10.3390/diagnostics15030316

**Published:** 2025-01-29

**Authors:** Mehrnaz Saghab Torbati, Ahmad Zandbagleh, Mohammad Reza Daliri, Amirmasoud Ahmadi, Reza Rostami, Reza Kazemi

**Affiliations:** 1 Neuroscience and Neuroengineering Research Laboratory, Biomedical Engineering Department, School of Electrical Engineering, Iran University of Science and Technology, Tehran 1684613114, Iran; mehrnaz.s.torbati@ieee.org (M.S.T.); a_zandbagleh@alumni.iust.ac.ir (A.Z.); 2 Max Planck Institute for Biological Intelligence, 82319 Seewiesen, Germany; amirmasoud.ahmadi@bi.mpg.de; 3 Department of Psychology, University of Tehran, Tehran 1445983861, Iran; rrostami@ut.ac.ir; 4 Department of Entrepreneurship Development, Faculty of Entrepreneurship, University of Tehran, Farshi Moghadam (16 St.), North Kargar Ave., Tehran 1439813141, Iran; rezakazemi@ut.ac.ir

**Keywords:** bipolar disorder, computer-aided diagnosis, EEG, explainable AI, Hjorth parameters, neurophysiological markers

## Abstract

**Background:** Despite the prevalence and severity of bipolar disorder (BD), current diagnostic approaches remain largely subjective. This study presents an automatic diagnostic framework using electroencephalography (EEG)-derived Hjorth parameters (activity, mobility, and complexity), aiming to establish objective neurophysiological markers for BD detection and provide insights into its underlying neural mechanisms. **Methods:** Using resting-state eyes-closed EEG data collected from 20 BD patients and 20 healthy controls (HCs), we developed a novel diagnostic approach based on Hjorth parameters extracted across multiple frequency bands. We employed a rigorous leave-one-subject-out cross-validation strategy to ensure robust, subject-independent assessment, combined with explainable artificial intelligence (XAI) to identify the most discriminative neural features. **Results:** Our approach achieved remarkable classification accuracy (92.05%), with the activity Hjorth parameters from beta and gamma frequency bands emerging as the most discriminative features. XAI analysis revealed that anterior brain regions in these higher frequency bands contributed most significantly to BD detection, providing new insights into the neurophysiological markers of BD. **Conclusions:** This study demonstrates the exceptional diagnostic utility of Hjorth parameters, particularly in higher frequency ranges and anterior brain regions, for BD detection. Our findings not only establish a promising framework for automated BD diagnosis but also offer valuable insights into the neurophysiological basis of bipolar and related disorders. The robust performance and interpretability of our approach suggest its potential as a clinical tool for objective BD diagnosis.

## 1. Introduction

Bipolar disorder (BD), often referred to as bipolar depression, is a complex mood disorder that causes significant shifts in a person’s mood, energy, activity levels, and concentration [1]. These mood swings can be severe, making it difficult for individuals to manage their everyday lives. BD is characterized by alternating episodes of depression and periods of unusually high or elevated mood, with each episode lasting anywhere from several days to weeks. Approximately 40 million people worldwide were living with BD as of 2019, based on estimates from the Global Burden of Disease Study [2]. While BD affects various aspects of personal, social, and cognitive functioning, the exact cause remains elusive [3].

Currently, the diagnosis of BD primarily depends on self-reported symptoms and evaluations by healthcare professionals, following guidelines outlined in advanced diagnostic manuals, including the Diagnostic and Statistical Manual of Mental Disorders, Fifth Edition (DSM-V) and the International Statistical Classification of Diseases and Related Health Problems, Eleventh Revision (ICD-11) [4]. While these methods are valuable, they have limitations, which can delay accurate diagnoses. People may struggle to recognize their symptoms, avoid seeking help due to stigma, or fail to distinguish BD from other similar conditions—all of which can result in missed or delayed diagnoses [5]. For instance, major depressive disorder (MDD) often mimics depressive episodes of BD, leading to the underdiagnosis of the manic or hypomanic states essential for a BD diagnosis [6]. This difficulty is compounded by the fact that individuals may not recognize or report hypomanic behavior, often due to a lack of insights associated with subthreshold symptoms of hypomania [7]. Similarly, borderline personality disorder (BPD) shares symptoms such as brief episodes of intense emotions—including irritability and euphoria—complicating differential diagnosis [8,9]. Additionally, attention-deficit/hyperactivity disorder (ADHD) may present with symptoms like hyperactivity and impulsivity, which overlap with manic episodes, further challenging diagnostic clarity [9].

There is growing evidence linking neurological changes to BD [10]. Studies using neuroimaging techniques, particularly comparing BD patients to healthy controls (HCs), have uncovered significant differences in brain structure and function [11]. One such tool, the electroencephalogram (EEG), measures the brain’s electrical activity and primarily reflects the synchronized activity of large populations of neurons. It provides an objective indicator of the brain’s functional state. EEG is particularly useful because it offers high temporal resolution, capturing real-time patterns of brain activity, making it superior to other physiological measures [12]. This capacity for real-time data makes EEG an essential tool for understanding brain function, detecting abnormalities, and supporting the diagnosis of mental health disorders [13].

Machine learning (ML), a branch of artificial intelligence (AI), has become a powerful tool for analyzing large datasets. ML uses sophisticated algorithms to uncover patterns and make predictions. These algorithms have shown great promise in analyzing EEG data to detect subtle signals that might be missed by human clinicians, thereby improving the accuracy of mood state classification and BD diagnosis [12]. In the context of classifying EEG signals, researchers have explored a range of classifiers, such as linear discriminant analysis (LDA), support vector machines (SVMs), k-nearest neighbor (KNN), logistic regression (LR), Bayesian classification (BC), and decision trees (DT), to improve classification accuracy, as discussed in related works [14].

In BD studies, significant attention has been devoted to the role of brain oscillations and their relationship to EEG frequency bands, with findings indicating that abnormalities in the gamma and alpha frequency bands may be associated with disruptions in the brain’s GABAergic system and thalamus [15]. However, researchers continue to debate whether these abnormalities stem from changes in brain wave frequencies or dysfunctions in specific brain regions. Some studies have attempted to leverage these EEG frequency band abnormalities to aid in BD diagnosis [11]. Recent advancements in ML have achieved approximately 90% accuracy in predicting BD using EEG data [11]. As EEG technology evolves, its potential as a reliable tool for BD detection in clinical practice is becoming increasingly viable. However, despite their promise, ML models are often described as black-box systems, where their decision-making processes are not fully transparent. This lack of interpretability can undermine trust, especially in sensitive fields like healthcare [16]. To address these challenges, explainable AI (XAI) has emerged to provide clearer insights into how ML models reach their conclusions [17]. For mental health professionals working with BD, XAI techniques like class activation maps (CAMs) [18], Shapley additive explanations (SHAP), and layer-wise relevance propagation (LRP) [19] can highlight the specific brain regions, time intervals, or frequency bands that most influence a model’s output. By increasing transparency, XAI builds trust in AI-powered diagnostic tools, ensuring their effective use in clinical practice. Integrating XAI with EEG-based BD detection enhances the interpretability of AI models and promotes their broader acceptance in mental health diagnosis [20].

This study presents a framework for differentiating BD from HC using EEG data. It investigates the Hjorth parameters, activity, mobility, and complexity, while employing ML algorithms to improve diagnostic accuracy. Additionally, the framework incorporates XAI techniques to highlight key EEG features that influence classification decisions. To ensure clinical applicability and robustness, leave-one-subject-out cross-validation (LOSOCV) is utilized to evaluate model performance.

### Related Works

A growing body of literature has explored the use of resting-state EEG to distinguish individuals with BD from HC participants, leveraging both traditional analysis and ML approaches [11,21]. For instance, a study by Kam et al. [22] demonstrated that BD patients exhibit higher power in the beta and gamma frequency bands compared to HC participants, alongside abnormalities in neural coherence patterns. Furthermore, Arikan et al. [23] investigated EEG differences between BD, BPD, and HC groups. While significant EEG differences were identified between HC participants and clinical groups, BD and BPD displayed overlapping patterns, with both disorders showing altered alpha and beta power.

ML models have been applied to enhance BD diagnostics using EEG features. Mateo-Sotos et al. [24] employed an extreme gradient boosting (XGB) approach, achieving 94% accuracy in differentiating BD from HC participants using several non-linear features. Another study [25] identified abnormalities in alpha activity, particularly in the frontocentral regions of the brain, as a potential biomarker for distinguishing BD in adolescents. Using spectral power and entropy measures, the study achieved high accuracy (95.8%) in classifying BD patients versus HC participants, further emphasizing the diagnostic potential of EEG in younger populations. Similarly, Wang et al. [15] utilized Welch periodograms to extract EEG frequency band features, such as power, mean, variance, and Shannon entropy, for diagnosing bipolar depression. They applied multiple classifiers, including SVM, LDA, and self-organizing maps (SOM). Among these methods, SOM outperformed others with an accuracy of 97.62%, sensitivity of 98.7%, and specificity of 97.02%, demonstrating the potential of entropy-based features in automated BD detection. Additionally, Montgomery et al. [26] explored synthetic datasets to train ML models for BD diagnosis, focusing on EEG parameters such as theta-alpha mean, beta frequency band mean, and coherence measures. The study validated the potential of computational approaches in simulating clinical conditions and achieved robust diagnostic accuracy of 92% using a multi-layer perceptron classifier. Recently, Ma et al. [27] examined non-linear features derived from EEG signals, including fractal dimension and entropy measures, to capture the complexity and randomness in EEG data. They found entropy-based features to be the most effective, achieving a classification accuracy of 95.74%, sensitivity of 93.68%, and specificity of 96.33%.

While studies employing ML have shown promising results, to the best of our knowledge, most lack the integration of XAI techniques, which are crucial for interpreting model decisions and ensuring clinical applicability. This study aims to address these gaps in the detection of BD by integrating ML models with XAI techniques. This combination enhances both the interpretability of the models and their predictive accuracy. Utilizing a proprietary clinical dataset comprising a diverse group of BD patients and HC participants, the study evaluates the performance of various ML classifiers trained with Hjorth parameters. The models’ reliability and accuracy are validated through LOSOCV. Additionally, XAI features are incorporated to assist clinicians in understanding the decision-making process, offering valuable insights into the factors contributing to BD.

The rest of this paper is structured as follows: Section 2 describes the materials and methods, including details of the dataset, the proposed method, XAI, the extracted feature set, feature selection, ML algorithms, and statistical tests. The results of the proposed method are presented in Section 3. Section 4 and Section 5 provide a detailed discussion of this work and the conclusion of this paper, respectively.

## 2. Materials and Methods

### 2.1. Participants and EEG Data Recording

The dataset includes 20 patients diagnosed with BD (mean age = 36.58 years, standard deviation (SD) = 11.94; 10 females) and 20 HC participants. The diagnosis of BD was conducted as the initial step by clinical experts at the Atieh Clinic Neuroscience Center in Tehran, Iran, using the Persian version of Structured Clinical Interview for DSM-5 Research Version (SCID-5-RV) [28]. Only individuals meeting the criteria for BD were included in this study. Consequently, this study does not include patients diagnosed with MDD or recurrent depression. Additionally, all participants diagnosed with BD either were not using any medication at the time of the study or had cooperated by following a drug washout period. The inclusion criteria for this study required the following: (i) outpatients aged 18 to 65 years diagnosed with BD based on a clinical interview adhering to DSM-V criteria and (ii) moderate to severe scores on the BDI-II (scores of 29 and higher). It is important to note that the Persian version of the BDI-II exhibits acceptable test–retest reliability (*r* = 0.74) and good internal consistency (α = 0.87) [29]. All healthy individuals were evaluated with the Symptom Checklist-90 (SCL-90), and no major psychological disorders were found in any of the participants. Exclusion criteria encompassed the presence of comorbid Axis I and II disorders, imminent risk of suicide, and history of substance abuse. All procedures involving human participants in this study adhered to the ethical standards of the institutional and/or national research committee. They were conducted in accordance with the 1964 Declaration of Helsinki, including its later amendments or comparable ethical standards. This study was approved under the ethical number NNRL-22649617 by the Iran University of Science and Technology.

Resting-state eyes-closed EEG signals were recorded using 201 Mitsar-EEG devices (Mitsar Ltd., St. Petersburg, Russia) at a sampling frequency of 500 Hz, with a 19-channel electro-gel sensor-based EEG cap according to the 10–20 international system.

### 2.2. Methodology

**Figure 1:**
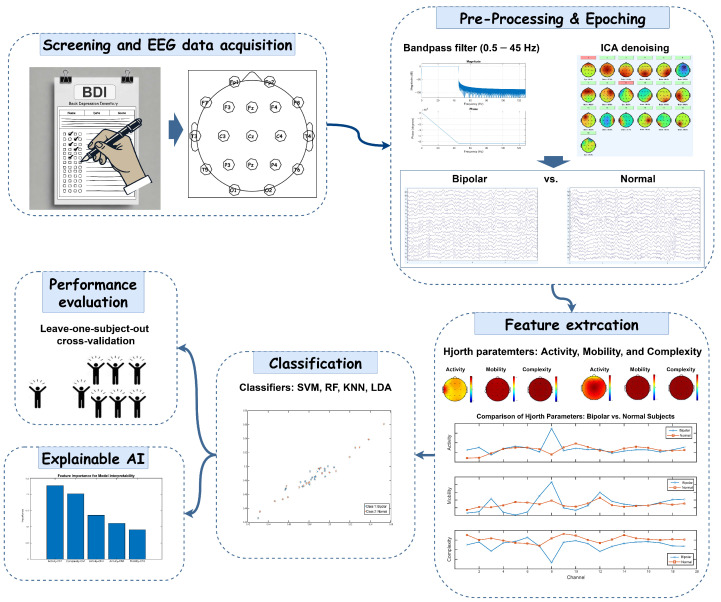
Proposed diagnostic framework for BD classification via EEG analysis. Nineteen-channel EEG signals were acquired from HC and BD patients. The selected segments underwent ICA-based preprocessing and epoching procedures. Feature extraction was performed using Hjorth parameters, and classification was implemented through LOSOCV methodology. The framework’s decision-making process was interpreted using XAI techniques to enhance model transparency and clinical relevance.

This study aimed to evaluate the robustness of time-domain EEG features as biomarkers for detecting BD using Hjorth parameters, combined with conventional ML classifiers and the XAI technique. A flowchart illustrating our proposed method is shown in Figure 1.

After screening individuals through clinical experts and questionnaires, and by recording resting-state EEG, preprocessing steps were applied to the raw EEG data. These steps included downsampling, band-pass filtering, and independent component analysis (ICA) for denoising. Consecutive epochs of 4 s were then extracted for further analysis. Then, Hjorth parameters, including activity, mobility, and complexity, were extracted as features and fed into some conventional classifiers. Subsequently, during the classification step, feature selection methods were implemented to enhance model robustness and speed by reducing the number of features. Performance evaluation was carried out using LOSOCV. Additionally, to enhance the reliability of our findings, we employed local interpretable model-agnostic explanations (LIME), an XAI approach, across all sets of characteristics. This method was used to pinpoint the most effective channels and features for the classification process. The application of XAI methods allowed us to improve detection and classification by emphasizing the most significant features.

### 2.3. Data Preprocessing

EEG data often contain noise due to various factors, particularly environmental interference, non-neural artifacts, and electrode impedance fluctuations, which can obscure meaningful brain signals and complicate subsequent analysis [12]. To enhance the reliability of bipolar detection, it is essential to mitigate this noise. In this study, several preprocessing steps were applied to the EEG data using EEGLAB [30] and its plug-ins to effectively reduce noise. The process began with downsampling the data to 250 Hz and applying a band-pass filter of 0.5 to 45 Hz, which effectively removed low-frequency artifacts and AC hum. Following this, manual noise reduction was performed to address noisy electrodes. The Common Average Reference (CAR) technique was then utilized to re-reference the EEG signals. Noisy timepoints were excluded through visual inspection. ICA was employed to separate and eliminate noise components, with the ICLabel plug-in facilitating the identification of non-EEG sources. Subsequently, any missing electrode data were interpolated using the spherical splines method. Finally, continuous EEG data were segmented into four-second epochs, which not only increased the sample size but also addressed the non-stationary nature of EEG signals [31]. This thorough approach ensured high-quality EEG signals for subsequent analysis.

### 2.4. Feature Extraction

Many scholars have conducted meaningful studies on EEG analysis and proposed several reliable features for the study of BD. Key features include alterations in linear measures, such as PSD [32], as well as non-linear measures [24], including the Hurst exponent, detrended fluctuation analysis, Higuchi fractal dimension, and various entropy measures. Additionally, connectivity metrics such as phase synchrony, synchronization likelihood, and coherence have been utilized to investigate the functional connectivity abnormalities frequently observed in individuals with BD [33]. This study investigates the altered activity across frequency bands in bipolar patients and examines how Hjorth parameters can enhance the differentiation between bipolar and normal EEG patterns. To achieve this, the Hjorth parameter feature extraction method was used to generate feature vectors for each 4 s segment. It is worth noting that Hjorth parameters are computed over five conventional sub-bands, including delta (0.5–4 Hz), theta (4–8 Hz), alpha (8–13 Hz), beta (13–30 Hz), and gamma (30–45 Hz). The following section discusses Hjorth parameters in detail.

- Hjorth Parameters

Hjorth parameters, also known as normalized slope descriptors, were introduced in 1970 [34] to measure signal complexity in the time domain using statistical calculations. Parameters such as activity, mobility, and complexity are widely used to analyze biological time series, particularly EEG signals, providing valuable insights into brain activity.

Hjorth parameters effectively characterize the time-domain properties of EEG signals. Activity measures the variance or power of a signal, representing its overall intensity or energy. A high activity value indicates greater signal variation, whereas a low value corresponds to more stable signals. This parameter is closely linked to the amplitude dynamics of EEG signals. Mobility is the second Hjorth parameter and measures the frequency characteristics of a signal. It is defined as the square root of the variance of the first derivative of the signal divided by the variance of the signal itself. This parameter indicates the speed or rate of signal changes, providing an insight into the dominant frequency of the signal. Complexity is the third Hjorth parameter and measures the degree of irregularity or intricacy of a signal. It represents how much the signal deviates from a simple sinusoidal waveform by comparing the mobility of the signal’s first derivative to the mobility of the signal itself [35]. These parameters can reveal differences in EEG dynamics associated with BD, highlighting variations in signal variability and complexity compared to HCs. Additionally, they enable meaningful data extraction from EEG signals without the computational burden of more complex methods, making them ideal for real-time analysis [36].

The proposed method for the time-domain analysis of EEG data involves deriving the autocorrelation function from polynomial coefficients. This function preserves all the information from power spectral analysis while also accounting for phase relationships between the contributing frequencies [36]. Time-domain analysis offers two key advantages: it allows for continuous calculations during real-time recordings and facilitates the interpretation of results in the physical context of the EEG source [36].

For the input signal x(n), the Hjorth parameter is calculated as follows:

(1) $$\mathrm{Activity}=\mathrm{Var}\left(x(n)\right)=\frac{1}{N}\sum_{n=1}^{N}{\left(x\left(n\right)-\bar{x}\right)}^{2}$$

(2) $$\mathrm{Mobility}=\sqrt{\frac{\mathrm{Var}\left(\frac{dx(n)}{dn}\right)}{\mathrm{Var}\left(x(n)\right)}}$$

(3) $$\mathrm{Complexity}=\frac{\mathrm{mobility}\left(\frac{dx(n)}{dn}\right)}{\mathrm{mobility}\left(x(n)\right)}$$

where x(n) and *N* are the input signal and the number of samples, respectively. Furthermore, $\bar{x}$ represents the average value, Var represents the variance, and $\frac{d}{dn}$ represents the first derivative of the input signal.

### 2.5. Feature Selection and Dimension Reduction

A substantial number of features are derived from the processing of EEG signals for each subject’s epoch, including five frequency bands, three Hjorth parameters, and 19 channels. To manage the high dimensionality of such datasets, feature selection methods are introduced. These approaches improve model accuracy, reduce redundancies, and enhance computational efficiency by decreasing training time and simplifying model complexity [37,38]. As researchers have previously mentioned, not all features are helpful for given applications [38]. Thus, focusing on crucial parameters, frequency bands, and informative EEG channels is essential for distinguishing between bipolar and healthy individuals. Removing less informative features can enhance the ability to capture brain dynamics associated with BD, leading to improved performance and interpretability.

There are several approaches used in the literature for feature selection and dimensionality reduction [37]. For instance, principal component analysis (PCA) achieves this by transforming features into new, linearly uncorrelated variables. In contrast, maximum relevance minimum redundancy (mRMR) directly selects a subset of the original features by maximizing their relevance to the target variable and minimizing redundancy. In this study, the mRMR method was used to further reduce the number of features and achieve optimal classification results [37]. Based on this approach, 12 features were identified as the best selection in this study. It is worth mentioning that prior probabilities for each class were specified as ‘empirical’, with other parameters set to their default values in the MATLAB ‘fscmrmr’ function.

### 2.6. Model Construction

This study employed a variety of well-established ML techniques to develop robust classification models with high precision. Classification modeling is a supervised learning approach in which a model is trained using labeled input data and subsequently evaluated on test data to measure its predictive accuracy [12,39]. Various classification methods are available, each offering unique advantages and potentially yielding distinct results. However, researchers have discovered that SVM often delivers superior classification results [40].

SVM is a robust classification algorithm that separates data in multi-dimensional space using kernel functions, with weighted scaling to enhance performance. Random forest (RF) improves the classification accuracy by constructing multiple decision trees on random sub-samples of the data and aggregating their predictions, thereby reducing the risk of overfitting. LDA is highly efficient for real-time applications, such as brain–computer interfaces, as it employs hyperplanes to classify data. However, its assumption of linearity can limit its applicability in non-linear scenarios. KNN classifies data based on proximity to its neighbors using distance metrics. While KNN is robust to noisy data, its performance heavily depends on the optimal selection of the number of neighbors [39].

By employing a variety of ML methods, we aimed to evaluate the consistency and robustness of the features across different classifiers.

### 2.7. Model Performance Evaluation

In the present study, the LOSOCV method was employed to assess the performance of the classification model, given the sample size limitation. LOSOCV involves using the data from N-1 participants for training and the remaining participants for testing. This process is repeated N times, with each participant’s data used once as the test set. By leveraging this approach, LOSOCV provides a more reliable estimate of the overall model performance. As such, classifier performance was evaluated using metrics such as accuracy, sensitivity, and specificity. Additionally, we applied the tuned model to the test data to generate receiver operating characteristic (ROC) curves and calculate the area under the curve (AUC), which serves as an indicator of model performance. Compared to accuracy alone, metrics such as ROC-AUC and confusion matrix parameters (sensitivity and specificity) offer a more comprehensive evaluation of performance.

### 2.8. Statistical Analysis

In this study, statistical analysis was performed to evaluate the diagnostic performance of Hjorth features extracted from different frequency bands. All analyses were conducted using MATLAB, with a significance level set at 0.05. Due to the non-normal distribution of the features, the Wilcoxon rank-sum test was employed to identify significant differences.

### 2.9. Explainable Artificial Intelligence

XAI is a branch of AI focused on making the decision-making processes of AI models transparent and comprehensible to humans. This transparency is particularly crucial in high-stake fields, such as healthcare, finance, and engineering, where critical decisions must be both reliable and well understood [41]. XAI addresses the black-box problem associated with complex models, such as deep learning, which often provide little insights into their reasoning. By utilizing techniques like feature attribution, surrogate models, and visual explanations, XAI clarifies how models reach their conclusions and highlights the features they consider most significant. LIME is one of prominent techniques in XAI, which explains individual predictions of black-box models [42]. LIME works by creating a simpler, interpretable model that approximates the behavior of the complex model locally, around a specific data point. By assigning weights to features based on their contribution to the prediction, LIME highlights the factors most responsible for the outcome. This enhanced clarity fosters trust, accountability, and adherence to ethical standards in AI applications. In both research and practical contexts, XAI plays a vital role in developing AI systems that are reliable and safe, ultimately promoting user confidence and transparency [41].

## 3. Results

All analyses in this study were performed on a Windows PC with an Intel^®^ Core™ i7-1065G7 processor (1.30 GHz) and 16 GB of RAM, using MATLAB 2024a. Preprocessing steps were carried out with EEGLAB v2024.0, and all other analyses were completed using custom MATLAB scripts and functions created specifically for this study.

In this study, EEG data were collected from 40 participants, equally divided between BD patients and HCs, with 50 epochs per participant, resulting in a total of 2000 epochs. Our analysis framework extracted 285 features from 19 electrodes, covering three Hjorth parameters (activity, mobility, and complexity) and five frequency bands (delta, theta, alpha, beta, and gamma). Following feature selection, 12 features were selected as the optimal set, yielding the highest accuracy. To ensure robust model evaluation, we implemented LOSOCV, where each iteration used 39 participants’ data for training while testing on the remaining participant. This approach allowed us to assess the generalizability of our feature selection methods and classification performance across different frequency bands, setting the foundation for our detailed analysis of discriminative EEG patterns between BD patients and HCs.

The classification performance results are presented in Table 1, Table 2 and Table 3. Table 1 presents the performance metrics of different classifiers analyzing Hjorth activity parameters across various EEG frequency band combinations for distinguishing between BD and HC participants. The linear-SVM classifier demonstrated superior performance, achieving the highest classification accuracy of 92.05% (sensitivity: 90.10%, specificity: 94%) in the beta–gamma band combination. This was closely followed by its performance in the delta–beta band, with 90.30% accuracy (sensitivity: 90%, specificity: 90.60%). The RF classifier showed strong performance in similar frequency bands, achieving 87.45% accuracy in beta–gamma and 88% in delta–beta combinations. While the LDA classifier maintained moderate performance levels, with its best results in the beta–gamma band (84.50% accuracy, 92.30% sensitivity, 76.70% specificity), it consistently underperformed compared to linear-SVM. The KNN classifier generally showed the lowest performance across most frequency bands, though it achieved notable accuracy in the beta–gamma band (85%). Notably, all classifiers demonstrated their strongest performance in the beta–gamma frequency band combination, suggesting this might be the most informative frequency range for distinguishing between BD and HC participants.

The analysis of Hjorth parameter combinations in the beta band reveals distinctive patterns in classification performance across different ML algorithms, as shown in Table 2. The experimental results demonstrate that parameter selection significantly influences classification accuracy, with certain combinations consistently outperforming others. Linear-SVM emerged as the most robust classifier, maintaining high accuracy across most parameter combinations, with its peak performance of 90.05% (sensitivity: 88.40%, specificity: 91.70%) being achieved using the activity–mobility combination. Interestingly, the addition of the complexity parameter did not substantially enhance performance, as evidenced by the marginally lower accuracy of 89.80% (sensitivity: 88.60%, specificity: 91%) when using all three parameters combined. The RF classifier showed particularly strong performance with the activity–complexity combination, reaching 86.80% accuracy (sensitivity: 84%, specificity: 89.60%), while its performance dropped notably to 77.45% with activity–mobility. The KNN classifier demonstrated consistent performance across different parameter combinations, achieving 85.05% accuracy (sensitivity: 80.40%, specificity: 89.70%) with activity–complexity and 82.55% (sensitivity: 75.70%, specificity: 89.40%) with activity–mobility. The LDA classifier maintained steady performance around 79% accuracy across most combinations, showing particular stability with the activity–mobility–complexity combination (79.85% accuracy, sensitivity: 82.40%, specificity: 77.30%). A striking observation across all classifiers was the markedly poor performance when using only the mobility–complexity combination, with accuracies dropping significantly, linear-SVM showing its lowest performance at 43.25%, while RF achieved the highest in this category at just 65.50%. This consistent pattern strongly suggests that the activity parameter plays a fundamental role in discriminating between the studied conditions, and its inclusion appears to be crucial for achieving reliable classification results.

Table 3 provides the classification performance of various classifiers for Hjorth parameter combinations in the gamma band. The linear-SVM classifier demonstrated the best performance overall, achieving the highest accuracy of 81.95% for the complete set of Hjorth parameters (activity–mobility–complexity), along with strong sensitivity (77.90%) and specificity (86.00%). Notably, it also achieved the top accuracy in the activity–complexity (83.80%) and activity–mobility (83.60%) combinations, with comparable sensitivity and specificity values. The LDA classifier showed consistent performance across combinations, achieving its highest accuracy (77.95%) in the activity–complexity combination, closely followed by activity–mobility (77.80%). When using all three parameters, its accuracy was 74.85%, slightly outperforming RF (74.50%) and KNN (74.35%) for the same combination. RF and KNN demonstrated similar performances across combinations. RF achieved its best accuracy (75.70%) for the activity–mobility combination, with balanced sensitivity (74.60%) and specificity (76.80%). Meanwhile, KNN’s highest accuracy (75.85%) was observed with the activity–complexity combination. For the mobility–complexity combination, performance across classifiers dropped significantly. Linear-SVM showed an accuracy of 53.00%, reflecting the challenge of using only these parameters. LDA, however, achieved relatively higher accuracy (64.65%), outperforming RF (56.15%) and KNN (57.80%). Overall, the results indicate that parameter combinations involving activity, either with mobility, complexity, or both, consistently yield better classification performance compared to other pairings. Linear-SVM emerged as the most effective classifier across these combinations, demonstrating a remarkable balance of high accuracy, sensitivity, and specificity. Its superior performance highlights its ability to exploit the full potential of the Hjorth parameters in distinguishing patterns in the gamma band. LDA, while not outperforming linear-SVM, showed reliable and competitive results, particularly in the activity–complexity combination, where it achieved its highest accuracy (77.95%) with sensitivity and specificity values that were close to linear-SVM’s performance. This suggests that LDA can be a robust alternative when computational simplicity is a priority. RF and KNN exhibited similar performance trends, achieving respectable accuracy levels but falling short of linear-SVM and LDA. Their performances were consistent across combinations, with RF performing slightly better in activity–mobility (75.70%) and KNN achieving its peak in activity–complexity (75.85%). These results demonstrate their effectiveness in moderately complex tasks but highlight their limitations in fully utilizing the discriminative power of Hjorth parameters. The mobility–complexity combination, on the other hand, presented notable challenges for all classifiers, resulting in significantly lower accuracies compared to other combinations. The accuracy of linear-SVM dropped to 53.00%, indicating that this parameter pairing lacks the discriminative features necessary for effective classification. Interestingly, LDA outperformed the other classifiers for this combination, achieving 64.65%, which highlights its resilience in scenarios where feature quality is limited.

In both the beta and gamma bands, incorporating activity consistently proved essential for robust classification using Hjorth parameters, whereas relying solely on mobility and complexity led to markedly lower accuracies. Across all tested classifiers, linear-SVM emerged as the top performer, achieving peak accuracy of 90.05% in the beta band (activity–mobility) and 81.95% in the gamma band (all three parameters). Notably, adding complexity to activity–mobility did not substantially boost performance in the beta band. LDA demonstrated reliable, competitive results, particularly for activity–complexity, often placing second behind linear-SVM and showing its resilience when feature quality was limited (e.g., outperforming other methods with mobility–complexity alone in the gamma band). RF and KNN exhibited moderate and similar performance trends, typically yielding accuracies in the mid-70% to mid-80% range across various combinations.

These findings highlight two key insights: (1) the Hjorth activity parameter plays a crucial role in distinguishing the studied conditions and (2) the linear-SVM classifier effectively leverages Hjorth parameters to achieve reliable, high-accuracy classification. Consequently, it is important to determine which EEG frequency band’s activity parameter offers the most effective biomarker for diagnosing BP. Table 4 summarizes the classification performance of various ML algorithms, using the activity parameter in different EEG sub-bands, by reporting the accuracy, sensitivity, and specificity for both BD and HC participants. Linear-SVM achieves the best overall performance, particularly in the beta sub-band, with an accuracy of 88.65%, a sensitivity of 86.60%, and a specificity of 90.70%. RF also performs well in the beta sub-band, showing similarly high metrics (accuracy = 87.75%, sensitivity = 84.10%, specificity = 91.40%). Although LDA and KNN achieve moderate results, they generally fall short of the performance levels demonstrated by linear-SVM and RF. These findings indicate that the Hjorth activity parameter, especially in the beta sub-band, may serve as a strong biomarker for identifying BD, given its consistently high accuracy, sensitivity, and specificity across classifiers.

**Figure 2:**
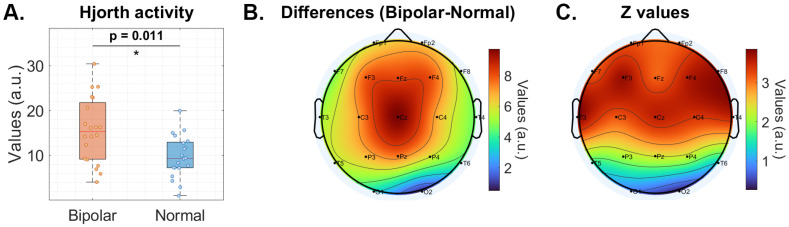
Distribution of Hjorth parameters for beta-band activity in healthy and bipolar subjects. (**A**) Mean Hjorth activity values for both groups. Asterisks (*) denote significant differences. (**B**) Channel-wise differences in Hjorth activity values between bipolar and healthy groups, where red areas indicate larger mean differences across all epochs and subjects. (**C**) Topographical map of z-values from a statistical comparison of Hjorth activity between bipolar and healthy participants.

Building upon the above classification insights, a closer examination of the Hjorth activity parameter in the beta frequency band reveals significant differences between BD and HC groups (Figure 2A). The BD group demonstrated consistently higher values in the activity parameter compared to the HC group within the beta frequency band. This elevated parameter suggests increased neural activation patterns and potential alterations in motor coordination characteristics associated with BD.

Topographical analysis (Figure 2B,C) of the spatial distribution of the Hjorth activity parameter in the beta frequency band across the scalp showed distinct patterns of difference between the two groups. The most pronounced differences in the beta activity parameter were observed in the frontal and frontocentral regions, particularly in electrodes F3 (*p*-value = $1.99\times 10^{-4}$, z-value = 3.72) and F4 (*p*-value = $3.38\times 10^{-4}$, z-value = 3.58). These channels exhibited significantly higher activity parameter values in BD patients compared to HC participants. The pronounced differences in Hjorth parameters in the frontal regions align with the established role of these areas in executive function and emotional regulation.

Figure 2B also shows the mean differences in beta activity; however, our analysis extends beyond these specific channels to investigate whether other brain regions also contribute to the diagnosis of high-performance BP. Table 4 presents the diagnostic performance of our model using Hjorth activity parameters across various frequency bands, demonstrating that the beta band alone achieves nearly 90% accuracy in detecting BP. Moreover, combining the beta and gamma bands boosts the diagnostic accuracy to 92.05% (Table 1). These findings underscore the critical importance of the beta and gamma bands as key biomarkers for BP, given that bipolar patients exhibit significantly higher activity levels in these frequency ranges, particularly in frontal channels, than healthy individuals.

**Figure 3:**
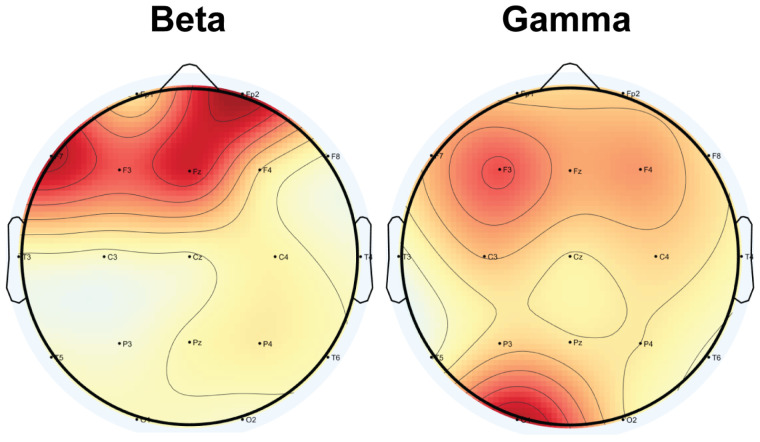
LIME analysis using an linear-SVM classifier for the beta and gamma frequency bands. Topographic maps display the importance values of Hjorth features across all EEG channels, as determined by LIME. Darker red areas indicate a greater contribution to the diagnosis of BD within each frequency band.

To elucidate the contribution of Hjorth activity parameters in our XAI-driven approach, we employed LIME techniques to assess how different brain regions influence the model’s ability to distinguish BP patients from controls. Figure 3 presents LIME-based visualizations of these influences, with areas in darker red signifying stronger contributions to the classifier’s decisions.

In the beta band, frontal channels emerged as the primary discriminators, with F4, Fz, F3, and right prefrontal (Fp2) channels showing especially high contributions. In contrast, the gamma band revealed a pronounced involvement of left frontal and central areas, as well as notable contributions from the occipital region (O1). The most influential channels were F3, O1, and F4.

Taken together, these LIME findings highlight distinct beta- and gamma-band signatures that differentiate BP patients from HCs. While beta-band activity in frontal regions supplies critical diagnostic information, gamma-band contributions are more localized to left-hemisphere frontal and occipital areas. This hemispheric asymmetry and frequency-specific distribution align with prior neurophysiological research on BD, underscoring the potential of frontal and occipital regions as key targets for future diagnostic advances and therapeutic interventions.

**Figure 4:**
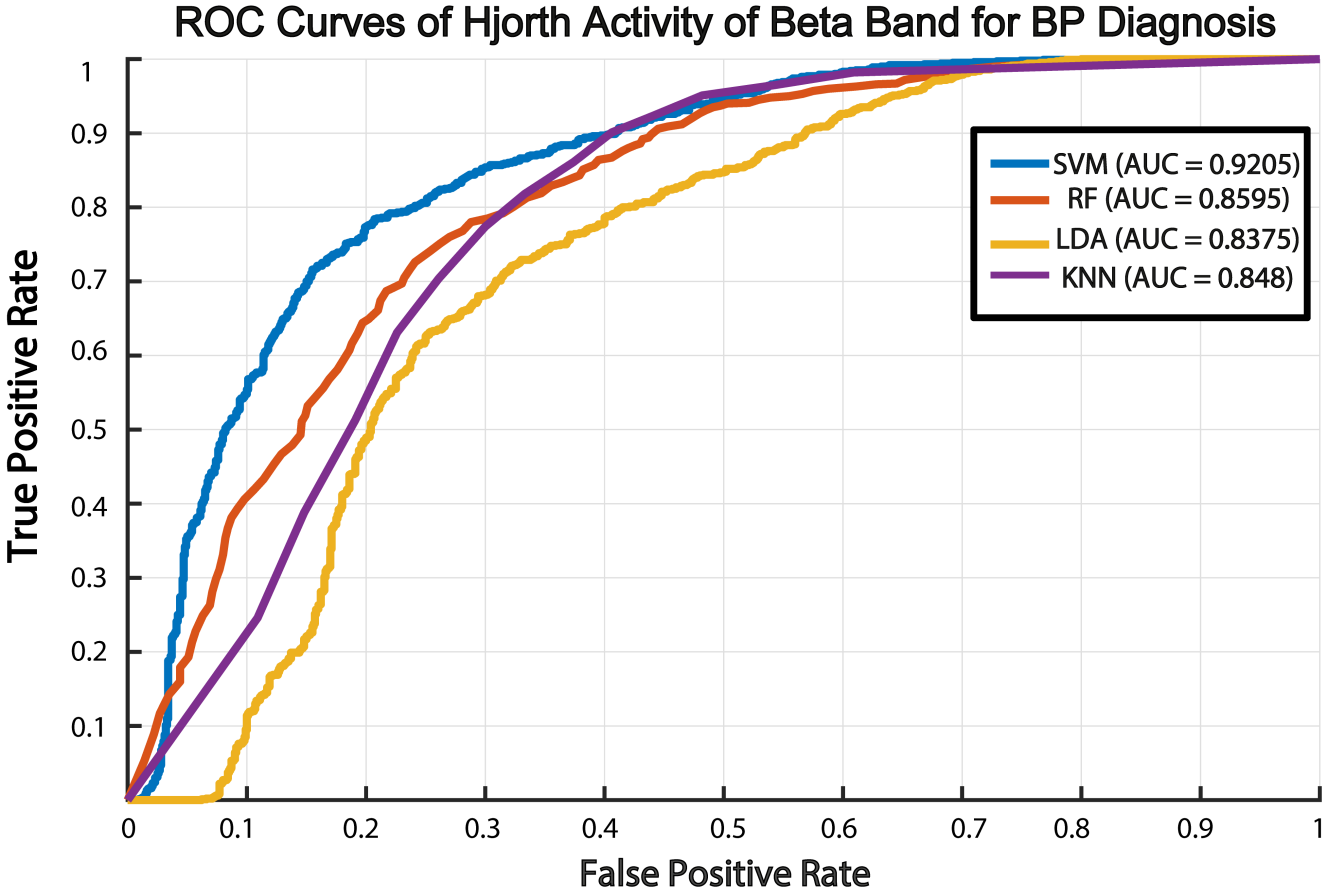
ROC curves and corresponding AUC values for various classifiers trained on activity Hjorth features in the beta frequency band.

To identify the optimal ML method for diagnosing BP, we performed a thorough feature selection and model comparison. Using mRMR, we narrowed down 12 key features that included Hjorth parameters (activity, mobility, and complexity) and spectral characteristics from the beta and gamma bands. These features were highly effective in distinguishing BD from HC participants. We then evaluated multiple ML classifiers, linear-SVM, KNN, LDA, and RF, to ensure robust model selection and address potential issues with generalization. Among these, the linear-SVM achieved the highest performance, with an AUC of 0.9205 in ROC analysis, followed by KNN and RF (Figure 4). We selected diverse classifiers due to their distinct strengths in handling varied data characteristics, aiming to demonstrate the importance of our features. Notably, the linear-SVM’s accuracy was highly sensitive to the number of features: when the feature set was reduced to 2, 4, or 6, performance declined, underscoring the importance of comprehensive feature selection. In particular, the linear-SVM model that utilized Hjorth activity and mobility features from the beta and gamma bands consistently outperformed the other classifiers. Validation via LOSOCV further demonstrated its robustness and generalizability, showing stable ROC-AUC values across validation folds.

## 4. Discussion

In this study, our objective was to investigate the potential of the EEG Hjorth parameters to distinguish between individuals with BD and HC. The main findings show that specific Hjorth parameters, particularly activity and mobility, demonstrated significant differences between BD patients and HCs, specifically in the beta and gamma bands. Notably, BD patients exhibited higher activity compared to HCs, indicating alterations in brain dynamics consistent with the literature on BD-related neurophysiological changes. These results align with previous studies reporting disturbances in brain oscillations of BD [25,32].

Increased cortical excitability is linked to high beta activity, which is generally thought to play a facilitating role [32]. The literature indicates that elevated emotional tension correlates with an increase in beta power, particularly in the anterior region [31,32].

The proposed method achieved its best performance by combining beta and gamma frequency bands. This combination outperformed all single-band cases and yielded accuracy, sensitivity, and specificity values of 92.05%, 90.10%, and 94%, respectively (using LOSOCV). Further statistical and XAI analyses revealed that anterior electrodes contributed the most to this classification (Figure 3). These findings regarding frequency bands and spatial contributions are consistent with previous studies on BD patients [32].

In our study, the beta band (13–30 Hz) achieved a higher classification accuracy (88.65%) compared to the gamma band (30–45 Hz), which showed 81.95%. This indicates the importance of the beta frequency band in BD patients [32].

LIME analysis identified the frontal lobes in the beta band and both the frontal and occipital regions in the gamma band as highly significant (Figure 3). These beta and gamma frequency bands in the anterior and occipital channels are critical for understanding and diagnosing BD. Within the frontal lobe, BD patients generally show increased beta and gamma activity compared to HCs. A previous study [43] has shown that BD patients exhibit deficits in gamma-band oscillations, which may be linked to dysfunction in GABAergic inhibitory interneuronal activity. The generation of gamma oscillations depends on synaptic GABA neurotransmission, which is essential for coordinating neural network activities across various brain regions. Cortical gamma activity plays an important role in processes such as sensory perception, memory, and problem solving [32].

In contrast, the occipital lobe normally exhibits a prominent alpha peak, but BD patients display abnormalities in both the beta and gamma frequency ranges. Occipital gamma activity is also relevant, though it manifests differently: when processing emotional faces, BD patients may show abnormal gamma responses indicating a hyperactive reaction to visual stimuli [44]. The observed increase in occipital beta power during manic episodes may function as a compensatory mechanism for impaired alpha responses [32]. Consequently, this elevation in beta-band power could be associated with a reduction in alpha activity [32].

To the best of our knowledge, few studies have applied XAI to BD patients using EEG data, particularly with a reliable validation method such as LOSOCV. The findings of this study demonstrate that XAI and statistical tests provide robust validation for the effective use of Hjorth parameters as a reliable criterion for identifying bipolar patterns. The results show that features extracted from frontal channels exhibit distinctiveness, which contributes to the development of a robust diagnostic system based on these features. Additionally, various established classifiers were employed to achieve optimal classification accuracy and highlight the importance of these features.

The initial phase of this study examined the effectiveness of Hjorth parameters in disease classification, supported by XAI results and statistical tests. As shown in Figure 3, the LIME analysis results emphasize that the frontal electrodes of the EEG play a crucial role in the classification process.

The findings of our research hold significant clinical implications. The ability to reliably classify BD from HCs using EEG signals could improve early diagnostic processes. The ML model, supported by the explainability provided by XAI techniques, such as LIME, offers clinicians transparency regarding the most influential features in decision making. This transparency is essential for building trust in AI-powered diagnostic tools, ensuring their safe and ethical integration into clinical practice. Furthermore, the observed changes in Hjorth activity may serve as potential biomarkers for BD. Clinicians could use these EEG features, in conjunction with traditional diagnostic methods, to gain deeper insights into the neurophysiological mechanisms underlying BD, leading to more accurate diagnoses. Moreover, this approach could be extended to help differentiate BD from other psychiatric disorders, such as MDD, schizophrenia, and obsessive compulsive disorder, which often share overlapping symptoms.

To demonstrate the effectiveness of our model, we compared its performance to a baseline accuracy of 50% (random chance for this balanced dataset) using the Binomial test to calculate *p*-values for each subject based on epochs. This approach evaluates whether the observed classification accuracy is significantly better than the expected accuracy under chance conditions. The application of the Binomial test supports its validity for assessing statistical significance in subject-specific analyses. The majority of *p*-values from our LOSOCV analysis of beta–gamma frequency band using linear-SVM are exceedingly small ($p<8.88\times 10^{-16}$ for 20 subjects), providing almost certain evidence that the model’s performance surpasses chance. A minority of *p*-values, such as $4.51\times 10^{-5}$, 0.00015, and 0.032, also remain statistically significant at conventional thresholds (p<0.05). However, two subjects yielded higher *p*-values (p>0.05), suggesting that for these individuals, the model’s performance was indistinguishable from chance. These outliers may reflect variability in subject-specific characteristics or other factors influencing classification outcomes. Overall, these results provide strong statistical support that the model performs significantly better than chance for the majority of subjects (38 out of 40, or 95% of subjects), confirming its genuine predictive power rather than random variation. If these subjects are considered valid representations, the accuracy and precision of the method become significantly more dependable, particularly in differentiating between BD and HC.

The findings of our study demonstrate that the linear-SVM algorithm is a highly effective tool for diagnosing BD, achieving an accuracy of 92.05%, with a sensitivity of 90.10% and specificity of 94%, using Hjorth activity parameters extracted from beta and gamma frequency bands. This result aligns with the range reported in similar studies, such as Khaleghi et al. [25], who achieved 95.8% accuracy using alpha-band EEG power and entropy features via a KNN classifier (k = 3), and Mateo-Sotos et al. [24], who reported 94% accuracy using an XGB classifier combined with linear and non-linear features. Additionally, another study [26] achieved 92% accuracy in BD diagnosis using an MLP classifier. A key difference between our study and those mentioned is the cross-validation approach; while most relied on k-fold or splitting methods, our study used the LOSOCV method, which offers a fundamentally different and more rigorous evaluation strategy.

Our study is distinguished by the rigorous use of the LOSOCV method. Unlike k-fold cross-validation, which risks overestimating performance, LOSOCV ensures that the model is tested on entirely unseen subjects, thereby closely mimicking real-world clinical conditions. This method has been rarely utilized, with one exception being a study by Wang et al. [15], which reported an accuracy of 97.62% using statistical features. Additionally, our incorporation of XAI offers significant advantages. While previous studies have employed conventional classifiers and a range of linear and non-linear features, their methods often lack interpretability. In contrast, our XAI approach identified the most discriminative features (Hjorth activity parameters in beta and gamma bands) and highlighted the role of anterior brain regions in BD detection. This provides valuable insights into the neurophysiological underpinnings of BD. Our model emphasizes interpretability and real-world applicability through LOSOCV, ensuring it remains both practical and clinically relevant. These results underscore the utility of beta and gamma frequency bands as biomarkers for BD, highlighting the potential of this framework as a reliable diagnostic tool.

The accuracy achieved in our study is consistent with the range reported in the literature [15,24,25,26,27], which varies from 85% to 97%, depending on the methodology and dataset. Studies employing advanced and complex features, such as multiscale entropy (Mateo-Sotos et al. [24]), have achieved comparable or higher performance, but require more computational time. In contrast, our proposed framework is computationally efficient due to its straightforward use of Hjorth features, making it more practical for clinical applications.

Despite these promising results and remarkable advantages, our study has some limitations. The relatively small sample size of 40 participants, although consistent with similar previous studies, limits the generalizability of our findings to broader populations. Another limitation of our study is the absence of a comparative group comprising individuals diagnosed with MDD. While our findings focus exclusively on individuals with BD, the inclusion of a comparative MDD group could offer additional insights into the differential characteristics and mechanisms underlying depressive episodes in these distinct clinical populations. Future studies could prioritize larger cohorts with more diverse sample sizes and incorporate advanced algorithms to enhance the assessment of depressive and related disorders. These efforts would contribute to a more comprehensive understanding of the nuances between BD and MDD in subsequent investigations. Additionally, while Hjorth parameters provide a computationally efficient and interpretable feature set, they may not fully capture the complexity of non-linear neural dynamics. Advanced feature extraction techniques, such as entropy-based or fractal analyses, combined with Hjorth activity features, could be explored to enhance sensitivity to subtle patterns in the data. Addressing these limitations will be crucial for refining diagnostic tools and ensuring their applicability in diverse clinical settings.

## 5. Conclusions

In this work, we introduced a novel framework that leverages XAI, Hjorth parameters, and LOSOCV to develop accurate, interpretable, and generalizable diagnostic tools for BD. By estimating multi-frequency band Hjorth parameters, our approach achieved a notable classification accuracy of 92.05%. This result underscores the effectiveness of integrating signal processing, XAI, and ML for identifying BD. Our findings reveal significant alterations in Hjorth activity, particularly in the beta and gamma frequency bands, when comparing BD patients to HCs, with the most pronounced differences observed in the frontal region. These results highlight the potential of Hjorth parameters as reliable biomarkers for BD detection and monitoring, enabling earlier intervention and reducing the risk of psychosis, particularly bipolar depression.

## Figures and Tables

**Table 1:** Hjorth activity classification results in different frequency band combinations. The highest and second-highest overall classification results are highlighted in bold.

Frequency Bands	Linear-SVM	RF	LDA	KNN
Delta–theta	AC = 68.25% SN = 66.50% SP = 70%	AC = 59.50% SN = 58.40% SP = 60.60%	AC = 61.50% SN = 47.35% SP = 75.60%	AC = 53.90% SN = 40.40% SP = 67.40%
Delta–alpha	AC = 67.40% SN = 70.10% SP = 64.70%	AC = 58.90% SN = 58.90% SP = 58.90%	AC = 64.90% SN = 54.70% SP = 75.10%	AC = 56% SN = 45.70% SP = 66.30%
Delta–beta	**AC = 90.30%** **SN = 90%** **SP = 90.60%**	AC = 88% SN = 85% SP = 91%	AC = 81.95% SN = 80.10% SP = 83.80%	AC = 72.25% SN = 62% SP = 82.50%
Delta–gamma	AC = 82.75% SN = 79.90% SP = 85.60%	AC = 75.20% SN = 75% SP = 75.40%	AC = 76.90% SN = 80.20% SP = 73.60%	AC = 54.65% SN = 43.30% SP = 66%
Theta–alpha	AC = 72.20% SN = 75.20% SP = 69.20%	AC = 61.65% SN = 65.70% SP = 57.60%	AC = 62.20% SN = 55% SP = 69.40%	AC = 57.50% SN = 53.50% SP = 61.50%
Theta–beta	AC = 89.30% SN = 86.60% SP = 92%	AC = 86.85% SN = 83.10% SP = 90.60%	AC = 76.80% SN = 75.90% SP = 77.70%	AC = 76.75% SN = 70.40% SP = 83.10%
Theta–gamma	AC = 83.95% SN = 84.10% SP = 83.80%	AC = 73.85% SN = 77.40% SP = 70.30%	AC = 74.20% SN = 82.10% SP = 66.30%	AC = 64.50% SN = 63.80% SP = 65.20%
Alpha–gamma	AC = 82.60% SN = 80.60% SP = 84.60%	AC = 74.15% SN = 73.70% SP = 74.60%	AC = 71.70% SN = 80% SP = 63.40%	AC = 58.95% SN = 56.20% SP = 61.70%
Beta–alpha	AC = 89% SN = 88.50% SP = 89.50%	AC = 79.05% SN = 76.70% SP = 81.40%	AC = 77.85% SN = 77.60% SP = 78.10%	AC = 72.50% SN = 68.60% SP = 76.40%
Beta–gamma	**AC = 92.05%** **SN = 90.10%** **SP = 94%**	AC = 87.45% SN = 82.80% SP = 92.10%	AC = 84.50% SN = 92.30% SP = 76.70%	AC = 85% SN = 79.60% SP = 90.40%

Abbreviations: SVM, support vector machine; RF, random forest; LDA, linear discriminant analysis; KNN, k-nearest neighbors; AC, accuracy; SN, sensitivity; SP, specificity.

**Table 2:** Hjorth parameter combination classification results in beta band. The best overall classification results in each Hjorth parameter combination are highlighted in bold.

Classifier	Activity–Mobility	Activity–Complexity	Mobility–Complexity	Activity–Mobility–Complexity
Linear-SVM	**AC = 90.05%** **SN = 88.40%** **SP = 91.70%**	**AC = 89.70%** **SN = 87.20%** **SP = 92.20%**	AC = 43.25% SN = 34.50% SP = 52%	**AC = 89.80%** **SN = 88.60%** **SP = 91%**
RF	AC = 77.45% SN = 74% SP = 80.90%	AC = 86.80% SN = 84% SP = 89.60%	**AC = 65.50%** **SN = 60.60%** **SP = 70.40%**	AC = 74.90% SN = 72.50% SP = 77.30%
LDA	AC = 79.65% SN = 80.90% SP = 78.40%	AC = 79.15% SN = 78.50% SP = 79.80%	AC = 60.40% SN = 58.70% SP = 62.10%	AC = 79.85% SN = 82.40% SP = 77.30%
KNN	AC = 82.55% SN = 75.70% SP = 89.40%	AC = 85.05% SN = 80.40% SP = 89.70%	AC = 60.80% SN = 51.10% SP = 70.50%	AC = 83% SN = 76.30% SP = 89.70%

Abbreviations: SVM, support vector machine; RF, random forest; LDA, linear discriminant analysis; KNN, k-nearest neighbors; AC, accuracy; SN, sensitivity; SP, specificity.

**Table 3:** Hjorth parameter combination classification results in gamma band. The best overall classification results in each Hjorth parameter combination are highlighted in bold.

Classifier	Activity–Mobility	Activity–Complexity	Mobility–Complexity	Activity–Mobility–Complexity
Linear-SVM	**AC = 83.60%** **SN = 80.40%** **SP = 86.80%**	**AC = 83.80%** **SN = 79.70%** **SP = 87.90%**	AC = 53% SN = 33.30% SP = 72.70%	**AC = 81.95%** **SN = 77.90%** **SP = 86%**
RF	AC = 75.70% SN = 74.60% SP = 76.80%	AC = 74.70% SN = 75.30% SP = 74.10%	AC = 56.15% SN = 51.70% SP = 60.60%	AC = 74.50% SN = 75.70% SP = 73.30%
LDA	AC = 77.80% SN = 79.80% SP = 75.80%	AC = 77.95% SN = 79.10% SP = 76.80%	**AC = 64.65** **SN = 58%** **SP = 71.30%**	AC = 74.85% SN = 79.40% SP = 70.30%
KNN	AC = 75.80% SN = 77.10% SP = 74.50%	AC = 75.85 SN = 77% SP = 74.70%	AC = 57.80% SN = 42.70% SP = 72.90%	AC = 74.35% SN = 75.20% SP = 73.50%

Abbreviations: SVM, support vector machine; RF, random forest; LDA, linear discriminant analysis; KNN, k-nearest neighbors; AC, accuracy; SN, sensitivity; SP, specificity.

**Table 4:** The Hjorth activity classification accuracy, sensitivity, and specificity for BD and HC participants in different EEG sub-bands using different classifiers.

Classifier	Delta	Theta	Alpha	Beta	Gamma
Linear-SVM	AC = 49.50% SN = 50.10% SP = 48.90%	AC = 69.10% SN = 64.10% SP = 74.10%	AC = 65.10% SN = 66.90% SP = 63.30%	AC = 88.65% SN = 86.60% SP = 90.70%	AC = 81.95% SN = 77.90% SP = 86%
RF	AC = 57.80% SN = 57.60% SP = 58%	AC = 58.45% SN = 62.30% SP = 54.60%	AC = 64.95% SN = 64.10% SP = 65.80%	AC = 87.75% SN = 84.10% SP = 91.40%	AC = 75.50% SN = 75.30% SP = 75.70%
LDA	AC = 61.95% SN = 40.20% SP = 83.70%	AC = 58.70% SN = 39.50% SP = 77.90%	AC = 65.05% SN = 59% SP = 71.10%	AC = 79.70% SN = 80.80% SP = 78.60%	AC = 77.55% SN = 80.60% SP = 74.50%
KNN	AC = 51.05% SN = 35.20% SP = 66.90%	AC = 52.95% SN = 50.90% SP = 55%	AC = 58.05% SN = 55.30% SP = 60.80%	AC = 82.50% SN = 77.50% SP = 87.50%	AC = 78.25% SN = 76% SP = 80.50%

## Data Availability

This study’s data can be made available from the corresponding author upon reasonable request. The data used in this study were preprocessed using the EEGLAB toolbox (https://sccn.ucsd.edu/eeglab/index.php, accessed on 1 April 2024) in MATLAB 2024a (MathWorks, Inc., Natick, MA, USA). Additionally, we were inspired by the methodology outlined in the All-in-One EEG Feature Extraction Toolbox (https://github.com/weizhenhit/All-In-One-EEG-Feature-Extraction-Toolbox, accessed on 1 April 2024) to develop our custom script for estimating Hjorth parameters. All other processing steps, including statistical analysis and machine learning, were performed using built-in MATLAB functions in MATLAB 2024a (MathWorks, Inc.). Custom scripts for these processes are available at our GitHub repository (https://github.com/Mehrnuz/Hjorth-EEG-Classification, accessed on 24 January 2025).

## Abbreviations

The following abbreviations are used in this manuscript:

AUC	Area under the curve
BD	Bipolar disorder
EEG	Electroencephalography
HC	Healthy control
ICA	Independent component analysis
KNN	K-nearest neighbor
LDA	Linear discriminant analysis

LOSOCV	Leave-one-subject-out cross-validation
ML	Machine learning
mRMR	Maximum relevance minimum redundancy
PCA	Principal component analysis
RF	Random forest
ROC	Receiver operating characteristic
SVM	Support vector machine
XAI	Explainable artificial intelligence
